# Impact of Secure Electronic Health Record Chat on Physician Communication in the Emergency Department

**DOI:** 10.5811/westjem.53220

**Published:** 2026-09-24

**Authors:** Michaela Go, Hasan Alshamrani, Obert Xu, Steven McGaughey

**Affiliations:** * Oregon Health and Science University, Department of Emergency Medicine, Portland, Oregon; † Oregon Health and Science University, Division of Informatics, Clinical Epidemiology and Translational Data Science, Portland, Oregon; ‡ King Abdulaziz University, Faculty of Medicine, Department of Emergency Medicine, Jeddah, Saudi Arabia

## Abstract

**Introduction:**

Effective communication in the emergency department (ED) is essential for safe patient care. Electronic health record (EHR)-integrated secure messaging may improve communication workflows, but limited evidence exists comparing clinician satisfaction across modalities in the ED. This study evaluated physician satisfaction with an EHR-integrated secure messaging tool compared to existing communication methods for non-emergent communication.

**Methods:**

We conducted an electronic pre-post survey study at an urban academic ED following implementation of an EHR-integrated secure messaging tool in October 2024. The primary outcome was overall communication satisfaction pre-post implementation. We also compared nursing communication satisfaction and repeat consult order proportions. Survey domains (ease of use, efficiency, timeliness, clarity, overall satisfaction) were graded using a four-point forced-choice Likert scale.

**Results:**

Response rates were 35% (33/95) pre-implementation and 36% (34/95) post-implementation. Although face-to-face communication maintained superior clarity ratings, secure chat received significantly higher overall satisfaction than phone communication (median 3 [IQR 3–4] vs 2 [2–3], *P* < .001) and higher ratings than the existing consult workflow across all domains (all *P* ≤ .03). Overall nursing communication satisfaction shifted toward higher scores post-implementation (median 3 [3–4] vs 3 [2–3], *P* = .03). Repeat consult order proportion decreased from 22% to 18% (risk difference, −3.5%; 95% CI, −4.5% to −2.5%; *P* < .001).

**Conclusion:**

Electronic health record-integrated secure messaging demonstrated high user satisfaction among emergency physicians and may reduce repeat consultant communications. Secure chat provides a complementary communication channel in the ED setting.

## INTRODUCTION

Effective communication and teamwork within the hospital setting are essential for high-quality patient care and preventing adverse events.^1^ Communication in the emergency department (ED) presents challenges due to variable patient volumes, high acuity cases, multitasking requirements, frequent interruptions, and decisions based on limited information.^2^–^4^ Traditional communication methods—face-to-face interaction, paging systems, and phone calls—each have limitations. Synchronous modes are associated with workflow interruptions,^5^ and paging systems entail delayed and unreturned responses.^6^ Face-to-face communication requires physically locating individuals, which is challenging in large hospital environments. Electronic health record (EHR)-integrated chat provides asynchronous communication that may reduce interruptions while maintaining message timeliness.^7^–^10^

Only two studies have evaluated asynchronous messaging in the ED, but neither compared satisfaction across modalities. Luu et al evaluated EHR-integrated secure messaging and found it reduced nonurgent calls and workflow interruptions,^11^ while Gulacti et al showed that WhatsApp, a third-party application, decreased ED length of stay and consultation time.^12^ In this study based on a survey of 95 emergency physicians, we addressed this gap through a pre-post implementation design comparing EHR-integrated secure messaging to existing communication modalities across multiple satisfaction domains in a large academic ED. In October 2024, our ED implemented EHR-integrated secure messaging as a supplementary tool for non-emergent communication. The study’s primary outcome was overall communication satisfaction. Secondary outcomes included domain-specific satisfaction scores, repeat consult order proportions, and interruption burden.

## METHODS

### Study Setting and Sample

We performed this study in an academic ED comprising 47 adult attendings, 15 pediatric emergency attendings, and 33 emergency medicine residents (*N* = 95). The ED has 42 licensed beds and approximately 60,000 patient visits annually.

### Communication Workflows

Emergency physicians communicate with nursing staff via department-issued phones and face-to-face interaction. Specialty consultations are initiated through EHR orders, which trigger paging through a centralized communications center that connects to the emergency clinician via department-issued phones. Follow-up communication with consultants requires placing repeat consult orders through the same workflow.

### Secure Chat Tool

Secure Chat (Epic Systems Corporation, Verona, WI) is an EHR-integrated messaging platform available on desktop and mobile devices with read receipts and delivery confirmation. Prior to implementation, institutional messaging established that Secure Chat was designated for non-emergent communication needs. It supplemented existing communication methods.

### Survey Development

The survey domains (ease of use, efficiency, timeliness, clarity, and overall satisfaction) were informed by constructs from a system usability scale, the DeLone and McLean Information Systems Success Model,^13^ and a technology acceptance model. We used a four-point forced-choice Likert scale (1 = very dissatisfied to 4 = very satisfied) to improve response polarity and reduce midpoint acquiescence bias.^14^ The survey instrument was reviewed by a departmental quality improvement committee and refined based on recommendations from an institutional survey methodology expert, including revisions to scale anchors and unipolar versus bipolar scale structure.^15^ Both surveys explicitly defined non-emergent communication as “messages that need to be communicated/asked in a timely manner but will not result in harm to the patient if not addressed immediately.” The instrument was pilot tested with practicing emergency physicians prior to distribution. Identical items and definitions were used in both surveys. The complete survey instruments are available as Supplemental Appendix A.

**Population Health Research Capsule**

What do we already know about this issue?

*Effective emergency department communication is essential for patient safety, but traditional methods like phone calls cause workflow interruptions and delays*.

What was the research question?

*Does electronic health record (EHR)-integrated secure chat improve physician satisfaction compared to existing communication methods?*

What was the major finding of the study?

*Secure chat had higher satisfaction than telephone communication (P < .001); the rate of repeat consults fell from 22% to 18% (P < .001)*.

How does this improve population health?

*Secure messaging may reduce communication barriers and workflow interruptions, supporting safer and more efficient patient care delivery*.

### Measures

Two anonymous surveys were distributed electronically via a cloud-based survey platform (Qualtrics International Inc, Provo, UT) to all 95 emergency physicians, representing a census of the eligible population. The pre-implementation survey assessed satisfaction with phone, face-to-face, and consult-page workflow communication. Three months after implementation of the EHR-based messaging tool, a parallel survey assessed Secure Chat. Both surveys assessed interruption frequency (1 = never to 4 = always) and disruptiveness (1 = not disruptive to 4 = extremely disruptive). We calculated response rates using the American Association for Public Opinion Research RR6 definition.^16^

We examined the proportion of repeat consult orders before implementation (July 1–September 30, 2024) and after implementation (November 1, 2024–February 28, 2025), excluding October for the uptake period. A repeat consult order was operationally defined as any subsequent order for the same consulting specialty within a single ED encounter, identified from an automated EHR report. This composite measure captures follow-up communications, non-responses requiring re-ordering, and duplicate entries.

### Statistical Analysis

Results are reported as median (interquartile range). We used Mann-Whitney U tests to compare satisfaction scores across communication modalities and to assess changes in satisfaction and interruption burden. We did not adjust for multiple comparisons; each comparison addressed a distinct, pre-specified research question rather than testing a universal null hypothesis, and correction methods such as Bonferroni increase Type II error risk in this context.^17^ Individual *P* values are reported to allow reader interpretation. Chi-squared test of independence evaluated changes in repeat consult proportions. We performed analysis using Python 3.12.7 (Python Software Foundation, Wilmington, DE).

### Institutional Review Board

The institutional review board classified this project as quality improvement not requiring its oversight.

## RESULTS

### Survey Response and Secure Chat Utilization

Response rates were 35% (33/95) for the pre-survey and 36% (34/95) for the post-survey. During the study period (November 2024–February 2025), emergency physicians sent 7,482 Secure Chat messages and received 13,595, confirming active tool adoption.

### Communication Satisfaction

Table summarizes satisfaction comparisons across all communication modalities and domains. Compared to face-to-face communication with nursing, Secure Chat received higher ease-of-use ratings (*P* = .04), although face-to-face communication maintained higher clarity ratings (*P* = .008). No significant differences were found for efficiency, timeliness, or overall satisfaction. Compared to phone communication with nursing, Secure Chat demonstrated significantly higher ratings for ease of use (*P* < .001), efficiency (*P* = .002), and overall satisfaction (*P* < .001). No significant differences were observed for clarity or timeliness.

For specialist consultant communication, Secure Chat showed significantly higher satisfaction than the existing consult workflow across all domains (all *P* ≤ .03; Table).

Overall satisfaction with nursing communication showed a significant shift in response distribution toward higher satisfaction after secure chat implementation (median 3 [IQR 3–4] versus 3 [2–3], U = 384.0, *P* = .03), with more physicians reporting “very satisfied” (12/34 [35%] versus 6/32 [19%]) (Figure).

### Repeat Consult Orders

The proportion of repeat consult orders was lower post-implementation (18%) compared to pre-implementation (22%) (χ^2^(1, *N* = 26,376) = 50.51, *P* < .001).

### Interruption Burden

Secure Chat interruptions were rated as less frequent than phone (U = 656.0, *P* = .03) and face-to-face interruptions (U = 679.5, *P* = .01). Secure Chat was also rated less disruptive than phone calls (median 2 [IQR 1–2] vs 3 [2–3], U = 850.5, *P* < .001) and face-to-face communication (median 2 [1–2] vs 2 [2–3], U = 701.5, *P* = .007). No significant changes in phone or face-to-face interruption patterns were observed after implementation.

## DISCUSSION

Secure Chat received higher nursing communication satisfaction ratings than face-to-face communication for ease of use, and it also received higher ratings than phone communication for ease of use, efficiency, and overall satisfaction. Secure Chat and phone received equivalent clarity ratings, although face-to-face communication remained superior for clarity, consistent with richer information exchange afforded by in-person interaction. For consultant communication, Secure Chat outperformed the existing consult page workflow across all satisfaction domains, likely reflecting friction inherent in the multi-step ordering process. Our findings align with prior research demonstrating high user satisfaction with secure messaging systems. Przybylo et al reported improved clinician satisfaction with a smartphone messaging system in a large hospital,^7^ and Gulacti et al demonstrated reduced consultation time with WhatsApp compared to phone communication.^12^ Consistent with the technology acceptance model, perceived ease of use showed the most consistent advantage for secure chat across all comparisons.

Secure Chat provides a complementary channel for non-emergent ED communications. Both surveys explicitly defined non-emergent communication, and institutional messaging prior to rollout established this boundary. The lower disruptiveness rating compared to phone and face-to-face communication suggests that asynchronous messaging may reduce workflow interruptions. The decrease in repeat consult orders suggests that Secure Chat may facilitate follow-up conversations that previously required re-ordering through the communications center.

## LIMITATIONS

Several limitations affect interpretation. The single-site design and three-month time frame limit generalizability and steady-state assessment. Response rates of 35–36%, while consistent with published ranges for electronic physician surveys,^16^,^18^,^19^ introduce the possibility of nonresponse bias. As a census rather than probability sample, findings should be interpreted with caution.

While the survey instrument was developed through expert review and established frameworks, it lacks formal psychometric validation. The four-point forced-choice scale may not capture the full range of respondent attitudes.^14^ The repeat consult metric is an indirect proxy that does not distinguish between orders placed due to nonresponse, duplicate entry, or clinical necessity, and the observed reduction cannot establish causation. We did not assess barriers to adoption, message-read rates, or the potential for missed asynchronous communications. Our study captured the perspectives of emergency physicians only; nursing staff, specialist consultants, and patient perspectives warrant future investigation. Future research should include multi-site designs, validated instruments, and longitudinal outcomes.

## CONCLUSION

Secure messaging integrated into the electronic health record showed high user satisfaction among emergency physicians for non-emergent communication and may reduce repeat consultant communications. While face-to-face communication remains superior for message clarity, Secure Chat provides a complementary communication channel in the ED setting.

## Supplementary Information



## Figures and Tables

**Figure:**
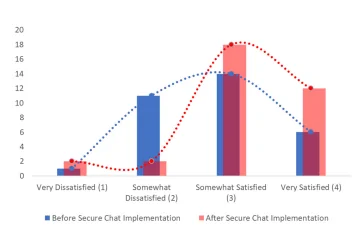
Overall satisfaction with nursing communication pre- vs post-Secure Chat implementation (all modalities combined) in a study of physician satisfaction with a secure messaging tool integrated into the electronic health record. Y-axis represents the number of respondents (pre, *n* = 32; post, *n* = 34). Scale: 1 = very dissatisfied, 2 = somewhat dissatisfied, 3 = somewhat satisfied, 4 = very satisfied. Mann-Whitney U test was used for comparison.

**Table:** Comparisons of communication by modality and domain in a survey-based study of emergency physicians’ satisfaction with a Secure Chat messaging tool integrated into the electronic health record.

Domain	Comparator Median (IQR)	Secure Chat Median (IQR)	U Statistic	*P* Value
Nursing: Secure Chat vs Face-to-Face (n = 34 vs n = 32)				
Ease of Use	3 (2–4)	4 (3–4)	392.0	.04*
Efficiency	3 (2–4)	3 (3–4)	420.5	.09
Timeliness	3 (3–4)	3 (3–4)	461.0	.26
Clarity	4 (3–4)	3 (3–4)	729.5	.008*
Overall	3.5 (3–4)	3 (3–4)	630.0	.23
Nursing: Secure Chat vs Phone (n = 34 vs n = 32)				
Ease of Use	2 (2–3)	4 (3–4)	231.5	< .001*
Efficiency	2.5 (2–3)	3 (3–4)	308.0	.002*
Timeliness	3 (3–4)	3 (3–4)	529.0	.84
Clarity	3 (3–4)	3 (3–4)	530.0	.85
Overall	2 (2–3)	3 (3–4)	293.0	< .001*
Consultants: Secure Chat vs Consult Workflow (n = 33 vs n = 29)				
Ease of Use	2 (2–3)	4 (3–4)	235.5	< .001*
Efficiency	2 (1–2)	4 (3–4)	168.5	< .001*
Clarity	3 (2–3)	4 (3–4)	335.5	.03*
Overall	2 (2–3)	3 (3–4)	224.0	< .001*

Scale: 1 = very dissatisfied, 2 = somewhat dissatisfied, 3 = somewhat satisfied, 4 = very satisfied.

Mann-Whitney U tests used for comparisons.

* Statistically significant (*P* < .05).

*IQR*, interquartile range.
