# Supplementary material for: Impact of Secure Electronic Health Record Chat on Physician Communication in the Emergency Department
Source: West J Emerg Med. 2026 Sep 24;27(5):1217–21. doi: 10.5811/westjem.53220 (PMC13660561; doi:10.5811/westjem.53220)
Supplement: Supplementary file 1 [file wjem-27-1217-s001.docx]

**Supplemental Appendix A**

**Survey Instruments**

*Evaluating the Impact of Secure Electronic Health Record Chat on Emergency Department Communication*

# Pre-Implementation Survey

*Distributed to all 95 ED physicians prior to Epic Secure Chat implementation.*

**Introduction text:** "Thank you for taking the time to participate in our survey! The purpose of this survey is to gain insights into the usage and perceptions of different communication methods within the emergency department and with specialty consultants. There are 7 questions. It will take about 3 to 5 minutes to complete. Data will be collected, de-identified, and used for quality improvement purposes."

## Question 1: Demographics

What is your role?

○ EM Resident PGY1 ○ EM Resident PGY2 ○ EM Resident PGY3 ○ Fellow ○ EM Faculty

## Questions 2–3: Communication with Nursing Staff

***Non-emergent messages: messages that need to be communicated/asked in a timely matter but will not result in harm to the patient if not addressed immediately.***

**Q2. Please rate your satisfaction with the following aspects of non-emergent communication conducted over the phone:**

| **Item** | **1 Very Dissatisfied** | **2 Somewhat Dissatisfied** | **3 Somewhat Satisfied** | **4 Very Satisfied** |
| --- | --- | --- | --- | --- |
| Overall Satisfaction | ○ | ○ | ○ | ○ |
| Easiness of use | ○ | ○ | ○ | ○ |
| Efficiency | ○ | ○ | ○ | ○ |
| Clarity of information received | ○ | ○ | ○ | ○ |
| Timeliness of information received | ○ | ○ | ○ | ○ |

**Q3. Please rate your satisfaction with the following aspects of non-emergent communication conducted face-to-face:**

| **Item** | **1 Very Dissatisfied** | **2 Somewhat Dissatisfied** | **3 Somewhat Satisfied** | **4 Very Satisfied** |
| --- | --- | --- | --- | --- |
| Overall Satisfaction | ○ | ○ | ○ | ○ |
| Easiness of use | ○ | ○ | ○ | ○ |
| Efficiency | ○ | ○ | ○ | ○ |
| Clarity of information received | ○ | ○ | ○ | ○ |
| Timeliness of information received | ○ | ○ | ○ | ○ |

## Question 4: Overall Nursing Communication

**Q4. How satisfied are you with non-emergent communication in the emergency department in general?**

| **Item** | **1 Very Dissatisfied** | **2 Somewhat Dissatisfied** | **3 Somewhat Satisfied** | **4 Very Satisfied** |
| --- | --- | --- | --- | --- |
| Overall Satisfaction | ○ | ○ | ○ | ○ |

## Question 5: Communication with Consultants

***Follow-up communication: Communication occurring after the initial call back from the consultant. Non-emergent: messages that need to be communicated/asked in a timely matter but will not result in harm to the patient if not addressed immediately.***

**Q5. Please rate your satisfaction with the following aspects of non-emergent follow-up communication conducted using the consult page workflow:**

| **Item** | **1 Very Dissatisfied** | **2 Somewhat Dissatisfied** | **3 Somewhat Satisfied** | **4 Very Satisfied** |
| --- | --- | --- | --- | --- |
| Overall Satisfaction | ○ | ○ | ○ | ○ |
| Easiness of use | ○ | ○ | ○ | ○ |
| Efficiency | ○ | ○ | ○ | ○ |
| Clarity of information received | ○ | ○ | ○ | ○ |

## Question 6: Overall Consultant Communication

**Q6. How satisfied are you with using consult page workflow for follow-up communication with consultants?**

| **Item** | **1 Very Dissatisfied** | **2 Somewhat Dissatisfied** | **3 Somewhat Satisfied** | **4 Very Satisfied** |
| --- | --- | --- | --- | --- |
| Overall Satisfaction | ○ | ○ | ○ | ○ |

## Questions 7–8: Interruption Assessment

**Q7. How frequently is your workflow interrupted by the following:**

| **Item** | **1 Never** | **2 Rarely** | **3 Sometimes** | **4 Always** |
| --- | --- | --- | --- | --- |
| Non-emergent phone calls | ○ | ○ | ○ | ○ |
| Non-emergent face-to-face communication | ○ | ○ | ○ | ○ |

**Q8. Rate the burden of disruptions to your workflow of the following communication modalities:**

| **Item** | **1 Not Disruptive** | **2 Minimally Disruptive** | **3 Moderately Disruptive** | **4 Extremely Disruptive** |
| --- | --- | --- | --- | --- |
| Non-emergent phone calls | ○ | ○ | ○ | ○ |
| Non-emergent face-to-face communication | ○ | ○ | ○ | ○ |

# Post-Implementation Survey

*Distributed to all 95 ED physicians three months after Epic Secure Chat implementation.*

**Introduction text:** "Thank you for taking the time to participate in our survey! The purpose of this survey is to gain insights into the usage and perceptions of different communication methods within the emergency department and with specialty consultants. There are 10 questions. It will take about 5–7 minutes to complete. Data will be collected, de-identified, and used for quality improvement purposes."

## Question 1: Demographics

What is your role?

○ EM Resident PGY1 ○ EM Resident PGY2 ○ EM Resident PGY3 ○ Fellow ○ EM Faculty

## Questions 2–3: Communication with Nursing Staff Using Secure Chat

***Non-emergent messages: messages that need to be communicated/asked in a timely matter but will not result in harm to the patient if not addressed immediately.***

**Q2. Please rate your satisfaction with the following aspects of non-emergent communication by Secure Chat:**

| **Item** | **1 Very Dissatisfied** | **2 Somewhat Dissatisfied** | **3 Somewhat Satisfied** | **4 Very Satisfied** |
| --- | --- | --- | --- | --- |
| Overall Satisfaction | ○ | ○ | ○ | ○ |
| Easiness of use | ○ | ○ | ○ | ○ |
| Efficiency | ○ | ○ | ○ | ○ |
| Clarity of information received | ○ | ○ | ○ | ○ |
| Timeliness of information received | ○ | ○ | ○ | ○ |

## Question 3: Overall Nursing Communication

**Q3. How satisfied are you with non-emergent communication in the emergency department in general?**

| **Item** | **1 Very Dissatisfied** | **2 Somewhat Dissatisfied** | **3 Somewhat Satisfied** | **4 Very Satisfied** |
| --- | --- | --- | --- | --- |
| Overall Satisfaction | ○ | ○ | ○ | ○ |

## Questions 4–5: Follow-up Communication with Consultants Using Secure Chat

***Follow-up communication: Communication occurring after the initial call back from the consultant. Non-emergent: messages that need to be communicated/asked in a timely matter but will not result in harm to the patient if not addressed immediately.***

**Q4. Please rate your satisfaction with the following aspects of non-emergent follow-up communication conducted using Secure Chat:**

| **Item** | **1 Very Dissatisfied** | **2 Somewhat Dissatisfied** | **3 Somewhat Satisfied** | **4 Very Satisfied** |
| --- | --- | --- | --- | --- |
| Overall Satisfaction | ○ | ○ | ○ | ○ |
| Easiness of use | ○ | ○ | ○ | ○ |
| Efficiency | ○ | ○ | ○ | ○ |
| Clarity of information received | ○ | ○ | ○ | ○ |

**Q5. How satisfied are you with using consult page workflow for follow-up communication with consultants?**

| **Item** | **1 Very Dissatisfied** | **2 Somewhat Dissatisfied** | **3 Somewhat Satisfied** | **4 Very Satisfied** |
| --- | --- | --- | --- | --- |
| Overall Satisfaction | ○ | ○ | ○ | ○ |

## Questions 6–7: Interruption Assessment

**Q6. How frequently is your workflow interrupted by the following:**

| **Item** | **1 Never** | **2 Rarely** | **3 Sometimes** | **4 Always** |
| --- | --- | --- | --- | --- |
| Non-emergent phone calls | ○ | ○ | ○ | ○ |
| Non-emergent face-to-face communication | ○ | ○ | ○ | ○ |
| Secure Chat Messages | ○ | ○ | ○ | ○ |

**Q7. Rate the burden of disruptions to your workflow of the following communication modalities:**

| **Item** | **1 Not Disruptive** | **2 Minimally Disruptive** | **3 Moderately Disruptive** | **4 Extremely Disruptive** |
| --- | --- | --- | --- | --- |
| Non-emergent phone calls | ○ | ○ | ○ | ○ |
| Non-emergent face-to-face communication | ○ | ○ | ○ | ○ |
| Secure Chat Messages | ○ | ○ | ○ | ○ |

## Questions 8–10: Secure Chat Evaluation (Post-Implementation Only)

**Q8. Secure Chat has improved communication in the ED:**

| **Item** | **Strongly Agree** | **Agree** | **Disagree** | **Strongly Disagree** |
| --- | --- | --- | --- | --- |
| (Single item) | ○ | ○ | ○ | ○ |

**Q9. What do you like about using Secure Chat for Communication in the ED?**

*[Open-ended free text response]*

**Q10. What do you dislike about using Secure Chat for Communication in the ED?**

*[Open-ended free text response]*

**Q11. Do you have suggestions for using Secure Chat effectively to improve ED communication?**

*[Open-ended free text response]*

## Response Scale Summary

| **Domain** | **Response Scale** |
| --- | --- |
| **Satisfaction** | 1 = Very Dissatisfied, 2 = Somewhat Dissatisfied, 3 = Somewhat Satisfied, 4 = Very Satisfied |
| **Interruption Frequency** | 1 = Never, 2 = Rarely, 3 = Sometimes, 4 = Always |
| **Disruption Burden** | 1 = Not Disruptive, 2 = Minimally Disruptive, 3 = Moderately Disruptive, 4 = Extremely Disruptive |
| **Improvement Agreement** | Strongly Agree, Agree, Disagree, Strongly Disagree |

**Note:** Both pre- and post-implementation surveys used identical items, scales, and definitions where applicable. The post-implementation survey included additional items (Q8–Q11) specific to Secure Chat evaluation. All satisfaction and interruption items used a 4-point forced-choice Likert-type scale with no midpoint option.
